# Revealing Medicinal Constituents of *Bistorta vivipara* Based on Non-Targeted Metabolomics and 16S rDNA Gene Sequencing Technology

**DOI:** 10.3390/molecules29040860

**Published:** 2024-02-15

**Authors:** Hui He, Chuyu Tang, Zhengfei Cao, Tao Wang, Min He, Mengjun Xiao, Licheng Xiao, Yuling Li, Xiuzhang Li

**Affiliations:** 1 State Key Laboratory of Plateau Ecology and Agriculture, Qinghai Academy of Animal and Veterinary Science, Qinghai University, Xining 810016, China; he15226330573@163.com (H.H.); chuyutang0410@163.com (C.T.); c1474477969@163.com (Z.C.); 13085500761@163.com (T.W.); himi1228@163.com (M.H.); 15574237597@163.com (M.X.); 2 Animal Husbandry and Veterinary Workstation in Yushu City, Yushu 815000, China; 15609768778@163.com

**Keywords:** endophytic bacteria, metabolites, *Bistorta vivipara*, root, medicinal components

## Abstract

*Bistorta vivipara* is a medicinal plant with a long history, but there are few studies on the effects of its medicinal components and endophytic bacteria on the accumulation of secondary metabolites. Therefore, in this study, non-targeted metabolomics techniques and 16s rDNA techniques were used to study *B. vivipara* from different regions. A total of 1290 metabolites and 437 differential metabolites were identified from all samples. Among them, flavonoids, isoflavonoids, and benzopyrans are the main medicinal components of *B. vivipara*; these have potential anticancer, antiviral, and antioxidant properties, as well as potential applications for the treatment of atrial fibrillation. In addition, irigenin, an important medicinal component, was identified for the first time. The endophytic bacterial communities in the root tissues of *B. vivipara* from different regions were also different in composition and richness. Hierarchical clustering heat map analysis showed that Proteobacteria and Actinobacteriota bacteria significantly affected the accumulation of many medicinal components in the roots of *B. vivipara*.

## 1. Introduction

*Bistorta vivipara* is one of the ectomycorrhizal plant species of *Bistorta*, Polygonaceae [1]. It has been used as a Chinese herbal medicine for hundreds of years. It is mainly distributed in alpine or subalpine meadows at an altitude of 1200–5100 m in Asia, Europe, and America [2,3]. Early studies have shown that changes in altitude can significantly affect its growth, development, and vegetative reproduction [4]. Its rhizome is considered to be primarily responsible for its pharmacological properties (e.g., antipyretic, antidiarrheal, menstruation-regulating, and hemostasis effects), and it is also an important food source for the host larvae of *Cordyceps sinensis* [5]. It has high medicinal, feeding, and ecological value [6].

As the growth environment of *B. vivipara* is harsh and the plant is difficult to collect, traditional research methods have some limitations. Therefore, there are few studies on its pharmacological activity, resulting in many potential medicinal values not being effectively developed. Some previous studies have identified volatile oils, organic acids, and sterols from *B. vivipara* via traditional methods. However, there are abundant primary and secondary metabolites in plants, which comprise only some of them [6]. Metabolomics technology is mainly used to identify functional secondary metabolites and metabolic pathways [7]. With the rise of this technology, further research on many medicinal components of plants, including *B. vivipara*, has become possible [8,9], which will contribute to the development of new clinical drugs. Metabolites are temporary structure–function complexes of sequential enzymes in metabolic pathways [10]. They are also the main components of many plants which exert biological activity [11], and they comprise a class of substances produced by plants in response to environmental changes for self-protection. They are mainly attributed to non-volatile compounds or volatile compounds, including flavonoids, peptides, and phenols [7,12]. Most of them are derived from the primary metabolites of plants, and they are widely involved in plant growth and development, innate immunity, environmental stress, and the avoidance of pests and diseases [7]. In previous studies, the terpenoid and phenylpropanoid metabolites extracted from the medicinal plant, *Ferula assafoetida*, were shown to exhibit anti-inflammatory and cytotoxic activities [13]. Ginsenosides can be extracted from many species of *Panax*, and these compounds have antioxidant, neuroprotective, and learning- and memory-enhancing properties in humans [14,15]. Chlorogenic acids (CGAs) are a class of naturally occurring polyphenols, widely found in medicinal plants such as *Eucommia ulmodies*, *Lonicera japonica*, and tea, which are known for their hepatoprotective effects [16,17]. Therefore, the metabolomic analysis of related plants is an effective way to reveal their medicinal components.

Endophytes are associated with most plant species in ecosystems. They are microorganisms that colonize plant tissues such as roots, stems, and leaves without causing adverse effects on the host plant [18,19,20,21]. They enter plants via microorganisms on the plant surface, plant rhizosphere, and environment through leaves, flowers, seeds, pollen, natural holes, and wounds, and they colonize for a long time, thus forming plant endophytes [21,22,23]. Some or all of these endophytes will exist in plant tissues, which, to some extent, affects the morphology and physiology of the plant [24,25]. During the long-term colonization process, endophytes form a wide range of mutually beneficial symbioses with the host plant [26]. They promote plant growth and development, and they improve plant nutrition or biosynthesis by secreting bioactive substances, such as 3-Indoleacetic acid (IAA), indole acetonitrile, gibberellin, and cell hormones such as plant hormone-like substances [20,21,26,27]. They are also involved in plant metabolism and hormone pathways, thus affecting the interactions between host plants, competitors, and pathogens [26]. Recent studies have further demonstrated their ability to reshape the micro-ecological characteristics of plants while being more favorable to photosynthesis in the host [28]. For example, studies on *Populus trichocarpa* showed that inoculation with root-endophytic bacteria significantly increased the root and leaf growth rates of the plant [24]. *Bacillus amyloliquefaciens* improves nutrient uptake, host plant growth and development, and hormone production in host plants [29]. Bacillus subtilis GbF-96, an endophytic bacterium from *Ginkgo biloba* L., showed significant antibacterial activity against five kinds of harmful foodborne pathogenic bacteria [30]. Therefore, understanding the diversity of endophytes in plants will help to improve plant health and thus facilitate the cultivation of some medicinal plants in captivity.

As an important component of the plant microecological environment, the plant endophytic flora significantly influences the synthesis and accumulation of plant-activating metabolites. Due to the long-term symbiotic relationship with the host, endophytes effectively avoid many harsh environmental factors and help plants absorb soil nutrients; they also resist the invasion of pathogenic fungi, which has great potential in improving plant health. Therefore, joint multi-omics analysis is an important tool with which to reveal the correlation between plant endophyte flora and metabolites [12,19,20,31]. A study focusing on endophytic bacteria and metabolites in *Ginkgo biloba* leaves by Fu et al. revealed the regulation of endophytic bacterial communities using flavonoid compounds and altitude changes [25]. Studies on endophytic actinomycetes in many medicinal plants have shown that the synthesis of many active compounds in plants has a significant correlation with these endophytes [32]. Endophytes, as microorganisms present in living plants, are one of the sources of many medicinal natural products, as are its host plants. For example, taxol, taken from a Taxaceae plant, has anticancer effects [33]. Endophytic bacteria, *Bacillus* spp., from many maize varieties, can secrete lipopeptide compounds that inhibit pathogenic fungi [34]. This shows that there is a close relationship between the endophytes and metabolites of medicinal plants. In this study, the diversity of endophytic bacterial communities and metabolites in the roots of *B. vivipara* was discussed, and a joint analysis was conducted to reveal the medicinal components of *B. vivipara*. The results of this study are expected to further improve the medicinal status of *B. vivipara*.

## 2. Results

### 2.1. Metabolomics Analysis of the Roots of Bistorta vivipara

**Figure 1:**
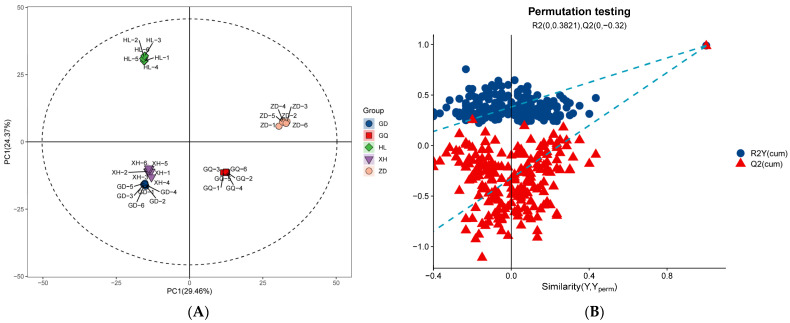
(**A**) Comparison of the first and second principal component scatter plots of PCA for each sample group. Each point represents a sample, and samples from the same group are represented by the same color. (**B**) OPLS-DA score plot of the sample group. The closer R2 and Q2 are to 1, the more stable and reliable the model; Q2 is greater than 0.5, indicating that the prediction ability of the model is good.

LC-MS/MS is a method that is widely used in plant metabolomics research [35]. In this study, LC-MS/MS technology was used to analyze the diversity of metabolites in the roots of *B. vivipara* from different sampling areas. A total of 1290 metabolites were detected in all collected root samples, including 818 positive ion mode (ESI+) metabolites and 472 negative ion mode (ESI−) metabolites (Table S1). The results of the principal component analysis (PCA) (Figure 1A) show that the five groups of samples had a high degree of dispersion, and the samples in the group were closely gathered together, indicating that the metabolites in each sample group exhibited high similarity. In the samples from the GD and XH sampling areas, the distance is closer, which may be caused by the similarity between metabolites and their content levels in the samples from the two sampling areas. The samples from the HL, GD, and XH sampling areas were all located on the negative X axis, whereas the samples from the GQ and ZD sampling areas were all located on the positive X axis, indicating that the metabolites of the *B. vivipara* roots, from different sampling areas, changed significantly depending on the region. In addition, the OPLS-DA model (Figure 1B) further showed that the metabolites between the sample groups were significantly different (*p* < 0.05).

### 2.2. Identification of Differential Metabolites in the Roots of Bistorta vivipara from Different Sampling Areas

**Figure 2:**
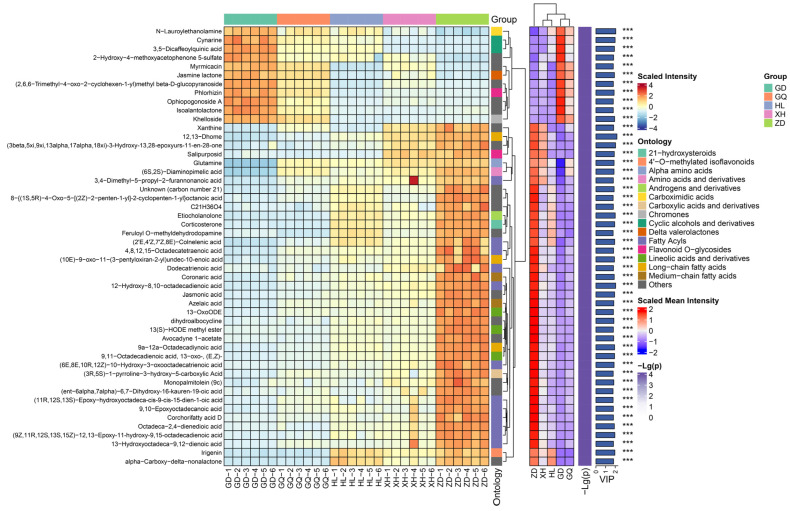
Complex heatmap of the hierarchical clustering of differential metabolites; comparison of sample groups (VIP top 50).

A total of 437 differential metabolites were screened from the identified 1290 metabolites, including 65 fatty acyls, 65 prenol lipids, 63 carboxylic acids and derivatives, 35 organooxygen compounds, 25 flavonoids, and 184 other metabolites (Table S2). The top 50 differential metabolites with variable importance for the projection (VIP) values were selected for abundance heat map analysis (Figure 2). The results show that the content of organooxygen compounds was higher in the samples from the GD and GQ groups, such as Cynarine, 3,5-Dicaffeoylquinic acid, and 2-Hydroxy-4-methoxyacetophenone 5-sulfate. The samples in the ZD group had higher levels of fatty acyls, such as jasmonic acid, azelaic acid, and 13-OxoODE. The contents of Etiocholanolone, irigenin, alpha-Carboxy-delta-nonalactone, and 12,13-Dhome in the HL and XH groups were significantly higher than those in the GD and GQ groups, but significantly lower than those in the ZD group. The metabolites in the sample groups were significantly different, indicating that the synthesis patterns of the secondary metabolites of *B. vivipara* growing in different regions may not be the same.

### 2.3. Differential Metabolite Functional Pathway Analysis

**Figure 3:**
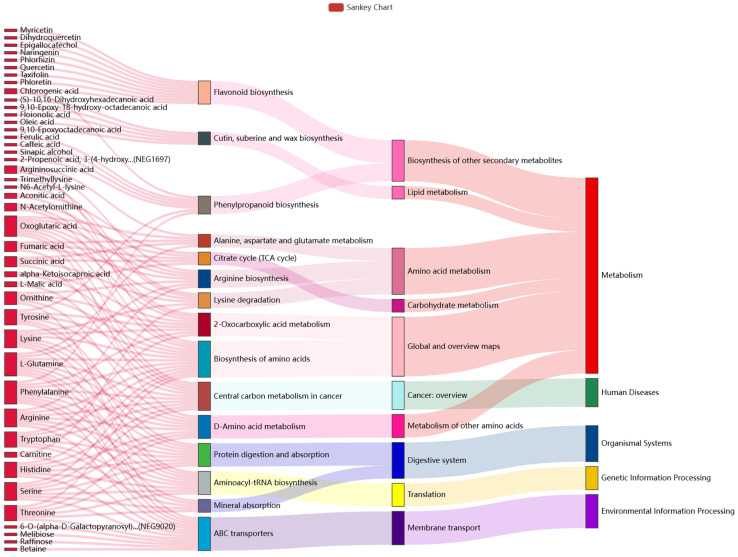
Sankey diagram comparing different groups. This describes the relationship between the top 15 pathways with enrichment significance and differential metabolites. The first column on the left represents the up-regulated or down-regulated differential metabolites. The height of the box indicates the number of metabolites annotated on the pathway; generally, the more corresponding pathways, the higher the box. The red flow line indicates the flow direction of up-metabolites, and the blue flow line indicates the flow direction of down-metabolites. The second, third, and fourth columns all indicate pathways, and the levels rise accordingly.

KEGG pathway analysis is the most direct and necessary way to understand the biological processes of cells, the mechanism of traits or diseases, and the mechanism of drug action in a more systematic and comprehensive manner. In this study, by referring to the KEGG database and previous studies, we analyzed the KEGG top 15 pathways of enrichment significance (Figure 3). The results show that a total of 43 differential metabolites were significantly enriched, and they showed an upward trend. Among them, ABC transporters, the Biosynthesis of amino acids, and the Central carbon metabolism in cancer, exhibited the largest numbers of enriched differential metabolites in the three KEGG pathways. In addition, Oxoglutaric acid, L-Glutamine, and Phenylalanine exhibited the largest numbers of annotated pathways, participating in 8, 9, and 9 metabolic pathways, respectively. Oxoglutaric acid is one of the marker metabolites that distinguishes between liver cancer and cirrhosis [36]. L-Glutamine can reduce the incidence of vascular occlusion crisis (VOC) [37]. Phenylalanine is an indispensable substance for patients with phenylketonuria [38].

### 2.4. Analysis of Endophytic Bacterial Diversity in Bistorta vivipara Roots

Dilution curves can reflect the sequencing depth of the samples. The results show that the curves tended to be flat, and the OTU coverage of each sample was close to 100% (Figure S1, Table 1), indicating that the sequencing depth could cover all species.

**Figure 4:**
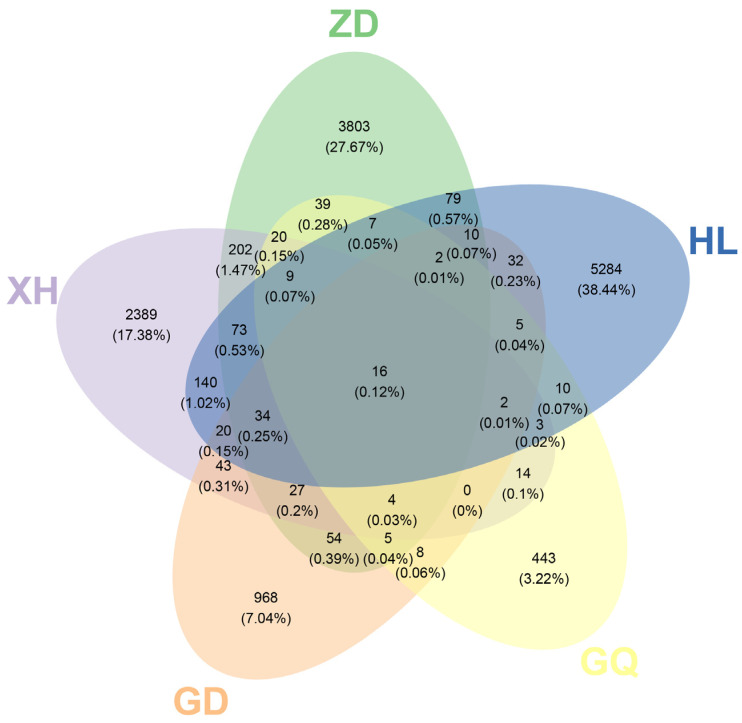
Venn diagram of the OTU distribution of endophytic bacteria in *B. vivipara* roots, which were collected from different sampling areas. Each ellipse represents a sample group (ZD, HL, GQ, GD, and XH), the overlapping region between ellipses indicates the shared OTUs between sample groups (ZD, HL, GQ, GD, and XH), and the number in each block indicates the number of OTUs contained in that block.

**Figure 5:**
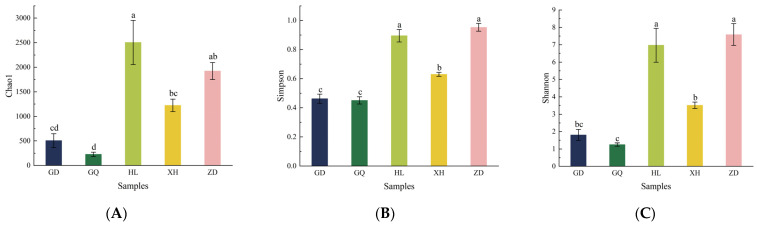
α-diversity index of endophytic bacteria in *B. vivipara* roots, which were collected from different sampling areas. (**A**) Chao1 index, (**B**) Simpson index, (**C**) Shannon index. GD, GQ, HL, XH, and ZD denote different sampling areas; For the same parameter, different letters above the bars denote a significant difference at *p* < 0.05.

The Venn diagram, which was constructed and based on the out, can be used to understand the composition of microorganisms in the sample. The results show that the HL sampling area had the highest number of endophytic bacterial OTUs in the root of *B. vivipara*, which accounted for 38.44%, whereas the GQ sampling area had the lowest number of endophytic bacterial OTUs in the root of *B. vivipara*, which accounted for 3.22% (Figure 4). The number of endophytic bacterial OTUs shared by the *B. vivipara* roots, which were collected from five different sampling areas, was extremely low, ranging from 0% to 1.47%, indicating that the composition of endophytic bacteria in *B. vivipara* roots, collected from different sampling areas, may be significantly different (Figure 4) [19]. The diversity and abundance of endophytic bacteria in the roots of *B. vivipara*, which were collected from different sampling areas, were analyzed using Chao1, Simpson, and Shannon indices (Figure 5, Table 1). The results show that the Chao1 index was highest in the HL sampling area, followed by the ZD sampling area, and lowest in the GQ sampling area. The results of Simpson’s and Shannon’s indices indicate that endophytic bacteria were similar in diversity and abundance in the HL and ZD sampling zones, as well as in the GD and GQ sampling zones.

**Figure 6:**
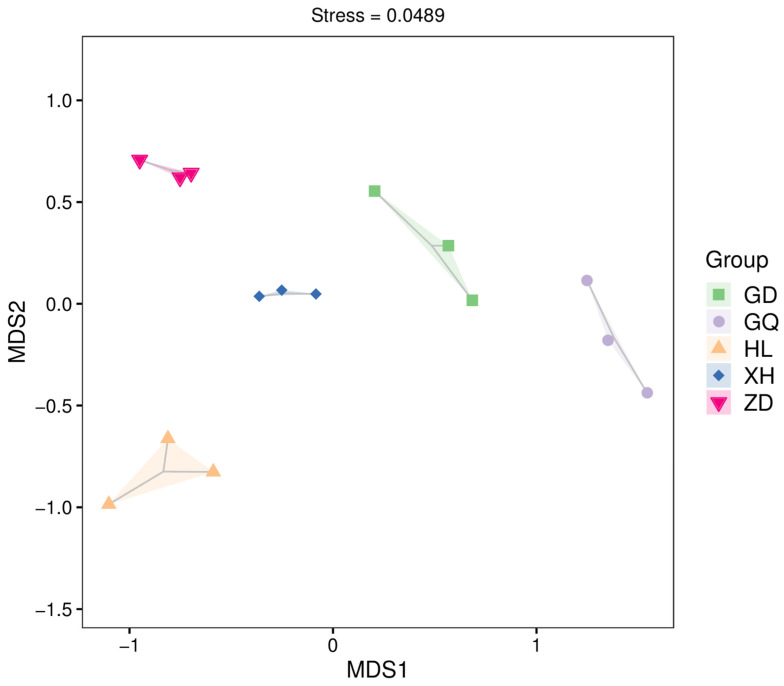
NMDS analysis of endophytic bacterial communities in the roots of *B. vivipara*, collected from different sampling areas. Each point on the graph represents a sample, with different colored points indicating different samples (groups), and the closer the distance between two points, the smaller the difference between microbial communities in the two samples.

Differences in the composition of endophytic bacterial communities in the roots of *B. vivipara*, collected from different sampling areas, were analyzed using NMDS at the OTU level (Figure 6). The results show a high degree of sample dispersion between the different sampling areas, indicating significant differences between the structures of endophytic bacterial communities in the roots of *B. vivipara* in different sampling areas.

### 2.5. Compositional Analysis of Endophytic Bacterial Communities in the Roots of Bistorta vivipara

**Figure 7:**
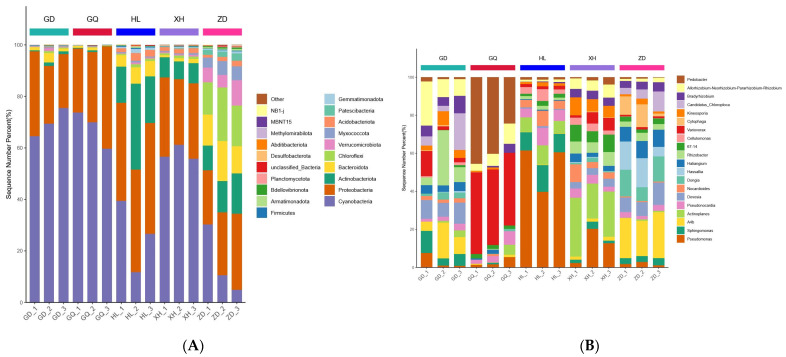
Diversity analysis of endophytic bacterial communities in the root of *B. vivipara* at the phylum and genus levels. (**A**) Abundance and composition of endophytic bacterial communities in the roots of *B. vivipara* at the phylum level. (**B**) Abundance and composition of endophytic bacterial communities in the roots of *B. vivipara* at the genus level.

A total of 38 phyla and 827 genera of bacterial OTUs were identified in all samples from different sampling areas. There were some differences between the relative abundances of endophytic bacterial communities in different sampling areas, but the composition was similar (Figure 7A,B). The highest abundances at the phylum level were related to Cyanobacteria, Proteobacteria, and Actinobacteriota, accounting for about 86% of the total abundance of endophytic bacterial communities in all samples. Among them, the relative abundance of Cyanobacteria in XH, GD, and GQ was significantly higher than that in HL and ZD (Figure 7A). The main role of Cyanobacteria involves a biological nitrogen fixation [7], and enrichment in the root may be more conducive to the growth of *B. vivipara* in the XH, GD, and GQ sampling areas. The dominant genera at the genus level are *Pseudomonas*, *Sphingomonas*, *A4b*, *Actinoplanes*, and *Pseudonocardia*. The richness of *Pseudomonas* and *Actinoplanes* in HL and XH was significantly higher than that in other sampling areas, whereas the difference between *Pseudonocardia* in different sampling areas was not significant (Figure 7B). It is suggested that the reconstruction of endophytic bacterial communities in plants growing in different regions may be a common phenomenon [35].

### 2.6. Predictive Analysis of Microbial Function

**Figure 8:**
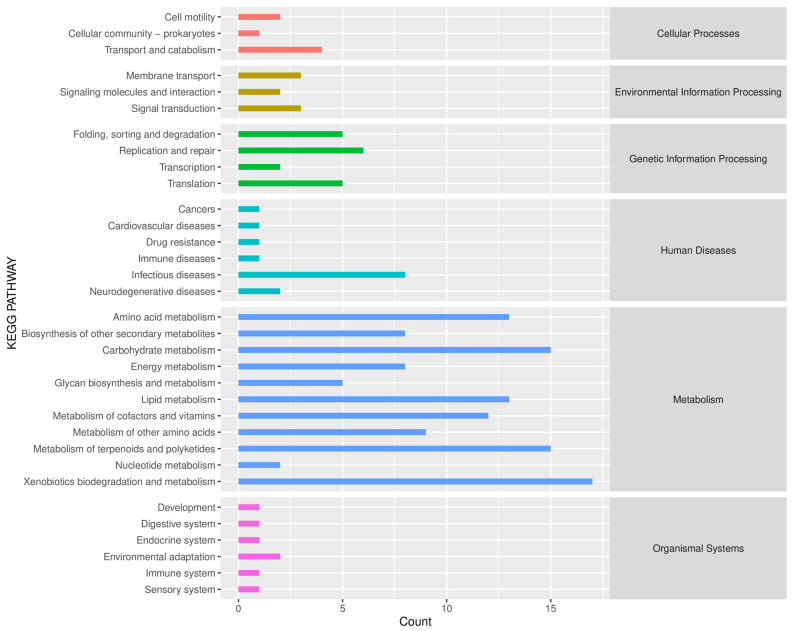
Predicted KEGG secondary functional pathway abundance map. The horizontal coordinate denotes the abundance of the functional pathway, the vertical coordinate denotes the functional pathway at the second classification level of KEGG, and the rightmost category denotes the first-level pathway to which the pathway belongs. This figure shows the average abundance of all samples.

Based on the KEGG path database, the Phylogenetic Investigation of Communities by Reconstruction of Unobserved States (PICRUSt2) was used to predict the function of endophytic bacteria in the roots of *B. vivipara* (Figure 8). The results show that the main functions of *B. vivipara* root endophytic bacteria include six categories, namely, Cellular Processes, Environmental Information Processing, Genetic Information Processing, Human Diseases, Metabolism, and Organismal Systems. Of these, the metabolism pathway accounts for the largest proportion. In addition, 33 metabolic pathways were identified in KEGG secondary functional pathways in all *B. vivipara* root samples. The top 10 were Xenobiotics biodegradation and metabolism, Metabolism of terpenoids and polyketides, Carbohydrate metabolism, Lipid metabolism, Amino acid metabolism, Metabolism of cofactors and vitamins, Metabolism of other amino acids, Infectious diseases, Biosynthesis of other secondary metabolites, and Energy metabolism. Nine of them belong to the Metabolism pathway and one belongs to Human Diseases.

### 2.7. Correlation Analysis of the Microbiomics and Metabolomics of Bistorta vivipara Root

**Figure 9:**
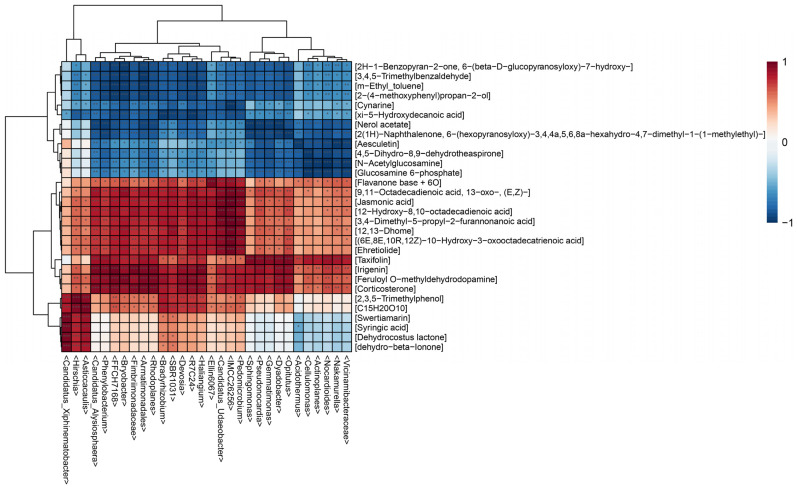
Hierarchical clustering heat map analysis. Rows indicate metabolites and columns indicate bacterial genera. The left tree branch represents the results of the clustering, which involved different elements of metabonomics, and the upper tree branch represents the results of the cluster analysis, involving the different elements of microbiomics. “***” in the small grid of the heat map indicates the correlation test, *p* < 0.001, “**” indicates the correlation test, *p* < 0.01, and “*” indicates the correlation test, *p* < 0.05.

In medicinal plants, specific microorganisms are involved in the biosynthesis of many important medicinal components. Therefore, in order to explore the complex relationship between endophytic bacterial communities and differential metabolites in the roots of *B. vivipara*, we conducted a correlation analysis between the top 30 endophytic bacterial genera and differential metabolites. The hierarchical clustering heatmap (Figure 9) shows that endophytic bacteria and differential metabolites were clustered into two categories, including 665 significant correlations. The results show that the endophytic bacterial community exhibited different assembly patterns in different sampling areas, and it made an important contribution to the grouping and quality-related factors of *B. vivipara*. Conversely, these differential metabolites may have the same effect on the endophytic bacterial community. Combined with the hierarchical clustering heat map (Figure 9), among all the differential metabolites positively related to the endophytic bacterial community, fatty acyls accounted for the largest proportion, which included jasmonic acid, Flavanone base +6O, 12,13-Dhome, and so on. The second group comprises prenol lipids, including Dehydrocostus lactone and dehydro-beta-Ionone. The differential metabolites which negatively correlated with the endophytic bacterial community were mainly benzene and substituted derivatives, including 3,4,5-Trimethylbenzaldehyde, m-Ethyl_toluene, and 2-(4-methoxyphenyl)propan-2-ol, as well as some fatty acyls, including xi-5-Hydroxydecanoic acid and Nerol acetate. It is evident that the endophytic bacterial community can regulate the accumulation of secondary metabolites, and the secretion and synthesis of secondary metabolites can also affect the composition of the endophytic bacterial community.

## 3. Discussion

A total of 1290 metabolites were identified using the root system of *B. vivipara* via LS-MS/MS, revealing the diversity of metabolites in the root system of *B. vivipara*. The differences between metabolites of *B. vivipara* in the roots, which were collected from different sampling zones, were revealed via PCA and OPLS-DA model analyses. These secondary metabolites were significantly different in different sampling areas. Organooxygen compounds, such as Cynarine, 3,5-Dicaffeoylquinic acid, and 2-Hydroxy-4-methoxyacetophenone 5-sulfate, were found at higher levels in the samples from the GD and GQ sampling areas. Fatty acyls, such as jasmonic acid, azelaic acid, and 13-OxoODE, were found at higher levels in the samples from the ZD sampling area. Etiocholanolone, irigenin, alpha-Carboxy-delta-nonalactone, and 12,13-Dhome were more abundant in the HL and XH samples. This shows that the synthesis patterns of the secondary metabolites of *B. vivipara*, collected from different sampling areas, are different; this may be caused by the influence of specific endophytic bacteria. In previous studies, many chemical constituents extracted from the tissues of the roots, stems, and leaves of host plants were shown to have important medicinal value, such as saponins, phenolic acids, etc. [19,20]. A study focusing on different metabolites in the root samples of *B. vivipara* showed that the metabolites with significant differences (and with respect to each sampling area) included many important medicinal components. These pharmaceutical ingredients are flavonoids, isoflavonoids, and benzopyrans. Flavonoids are some of the main components of various plant extracts [20], with antitumor, antioxidant, antiviral, and rhinitis relief effects [39,40,41]. Isoflavonoids play an important role in the prevention of type 2 diabetes, the prevention and mitigation of kidney disease and cardiovascular disease, and the inhibition of prostate cancer cell proliferation [42,43]. Benzopyrans have therapeutic potential for atrial fibrillation [44]. Previous studies on the chemical constituents of *B. vivipara* mainly focused on the antioxidant activity of 18 secondary metabolites [45]. This study further revealed the diversity of its medicinal components, which is conducive to the further development of the many potential medicinal components of *B. vivipara*.

In addition, we performed KEGG pathway analysis to reveal the differential pathways of *B. vivipara* from different sampling areas. The results show that the differential metabolites were mainly enriched in the ABC transporters, the Biosynthesis of amino acids, and the Central carbon metabolism in cancer KEGG pathways. Among them, 14 differential metabolites were enriched during the Biosynthesis of the amino acids pathway, including many active secondary metabolites; for example, histidine, which has a protective effect on the skin [46], and lysine, which can regulate hypertension and arrhythmia after acetylation [47]. In addition, in the second layer of the KEGG pathway, we found that in addition to the Global and overview maps pathway, the Amino acid metabolism pathway accounted for a large proportion, including Lysine degradation, Arginine biosynthesis, and Alanine, aspartate and glutamate metabolism. Amino acids, as a valuable biological resource, are an integral part of proteins and organisms, and they play a key role in regulating nitrogen balance in cells [48]. In previous studies, they have been found to improve muscle performance [49]. Diseases such as lupus and cancer are closely related to amino acid metabolism. Targeting amino acid metabolism and metabolic reprogramming could be potential therapeutic strategies [50,51]. This provides a potential basis for the development of the medicinal value of *B. vivipara*.

An endophyte is a kind of non-pathogenic microorganism existing in plant tissues. It maintains a co-evolutionary symbiotic relationship with the host plant, and it can enhance the host plant’s ability with respect to drought resistance, pest resistance, and salt tolerance [52,53,54]. In this study, the endophytic bacterial diversity in the roots of *B. vivipara* varied among five different sampling areas. Among them, the highest level of endophytic bacterial community diversity was found in the HL sampling area. Previous studies on *Kalidium foliatum* [55] and *Arachis hypogaea* [56] have shown that differences in the diversity of endophytic bacteria in the same plant, in different regions or different growth environments, is a common phenomenon [22]. The reasons for these differences are usually related to the biodiversity of the host plant habitat, the health status of the host plant itself, and the growth environment (temperature, drought, etc.) of the host plant [22]. The samples of *B. vivipara* we collected were similar in terms of health status and growth environment. The HL sampling area was sparsely populated, and the diversity of wild animals and plants was higher than in other sampling areas. This may be an important reason why the diversity of endophytic bacteria in the HL sampling area was higher than that in other sampling areas. In addition, the results of NMDS analysis show that the samples were clustered into five categories based on the sampling area, indicating that there were significant differences between the endophytic bacterial community structures of *B. vivipara* samples in different sampling areas.

The results, with respect to endophytic bacterial community diversity at the phylum level, show that the root tissues of *B. vivipara* were enriched with Cyanobacteria, Proteobacteria, and Actinobacteriota, which is similar to the root endophytic bacterial composition that has been reported for *Panax notoginseng* [15]. Cyanobacteria, Proteobacteria, and Actinobacteriota comprise a class of bacterial communities that are ubiquitous in plant tissues or in alpine grassland soils [15,57,58], which may be related to their strong environmental adaptability. Many bacteria from Actinobacteriota are considered to be saprophytic Gram-positive bacteria [59], which can enhance the ability of plants to resist drought stress by synthesizing nitric oxide with volatile organic compounds and producing phytohormones under drought conditions [60]. They can also degrade organic polymers such as lignin and suberin in plants [61,62]. Therefore, enrichment in host plants may affect the synthesis of some phenolic metabolites, but also improve plant resistance and promote plant growth [63]. Cyanobacteria and Proteobacteria comprise a class of Gram-negative bacteria with strong nitrogen fixation abilities. Enrichment in the roots of *B. vivipara* may affect the biosynthesis of nitrogen-containing substances such as alkaloids and proteins [19,63,64]. In addition, the richness of bacterial communities such as *Bradyrhizobium, Haliangium,* and *Solirubrobacter*, in the roots of *B. vivipara*, in different sampling areas, was significantly different. This may be related to their ecological adaptability or response to environmental changes. Therefore, by adjusting the species and content of endophytic bacterial communities in the roots of *B. vivipara*, the endophytic microbial community can reach a level conducive to plant growth.

PICRUSt is a technique for predicting macro genomes, based on 16S data, and reference genome databases, with high accuracy when conducting functional predictions, and it is now widely used for the functional prediction of endophytic bacterial communities in plants [19,65,66]. The functions of the endophytic bacteria in *B. vivipara* roots were classified into six main groups (i.e., Cellular Processes, Environmental Information Processing, Genetic Information Processing, Human Diseases, Metabolism, and Organismal Systems). Among them, the metabolism pathway accounted for the largest proportion. These results are similar to the results of previous studies which focused on *Panax quinquefolius* [19]. Since there are still some limitations to PICRUSt function prediction, the predictive abilities of the endophytic bacteria function may be preliminary. However, the research results can still provide an important reference for understanding the composition of the root endophytic functional bacterial community of *B. vivipara* and the excavation of the root endophytic functional bacterial community.

Previous studies have shown that there are extremely complex correlations between plant endophytes and plant metabolites [19,64]. More specifically, the transformation of host plant metabolites may be regulated by endophytes, which further influences their medicinal composition [15]. For example, the endophytic bacterium, *Bacillus altitudinis* LB 5-3, from *Panax ginseng*, can induce the accumulation of ginsenoside in adventitious roots as an elicitor [67]. The endophytic bacteria, *Ruminococcaceae bacterium* GD7 and *Mesorhizobium mediterranenum*, in the roots of *Paeonia lactiflora*, may be the key flora affecting the synthesis of two types of medicinal compounds (i.e., phenolic acids and flavonoids) [20]. In this study, endophytic bacteria were positively or negatively correlated with most of the differential metabolites. To further explore the relationship between endophytic bacterial communities and differential metabolites in the roots of *B. vivipara*, of the top 30 bacterial communities and differential metabolites in terms of abundance, we focused on the differential metabolites that were positively correlated with the root endophytic bacterial communities. Among these differential metabolites, jasmonic acid, taxifolin, and irigenin are the most important medicinal components in many medicinal plants; this has been confirmed in previous studies. Jasmonic acid is a fatty acyl compound with anticancer, anti-inflammatory, and cosmetic effects [68]. Taxifolin is a class of flavonoids that have antioxidant, anti-inflammatory, antibacterial, and bone loss prevention effects [69,70]. Irigenin is a class of isoflavonoids, which has mainly been derived from *Belamcanda chinensis* in previous studies, and it has important medicinal values, including antioxidant, anti-inflammatory, and antitumor properties [71]. In the present work, irigenin, an important compound, was identified using the root tissue of *B. vivipara*; this is the first time this has been done for *B. vivipara*. This further demonstrates the medicinal value of *B. vivipara*. Combined with non-targeted metabolomics and 16s rDNA sequencing technology, jasmonic acid, taxifolin, and irigenin were significantly positively correlated with bacterial communities at the 16, 18, and 20 genus levels, respectively (*p* < 0.001). These endophytic bacterial communities were mainly attributed to Proteobacteria and Actinobacteriota. In previous studies, Proteobacteria and Actinobacteriota were strongly associated with many plant secondary metabolites, including fatty acyls and flavonoids. This study further confirmed this result [35]. The results indicate that the content levels of the main medicinal components of *B. vivipara* may be greatly affected by the bacterial communities, with respect to Proteobacteria and Actinobacteriota.

## 4. Materials and Methods

### 4.1. Sample Collection

The sampling areas are located in Qinghai Province, China: Hualong Hui Nationality Autonomous County in Haidong City (36°13′20″ N, 102°19′54″ E), Zaduo County in Yushu Tibetan Autonomous Prefecture (32°47′38″ N, 95°8′23″ E), Xunhua Salar Autonomous County in Haidong City (35°34′30″ N, 102°44′23″ E), Guoqing Ranch in Yushu Tibetan Autonomous Prefecture (33°1′48″ N, 96°48′20″ E), and Guide County in Hainan Tibetan Autonomous Prefecture (36°8′21″ N, 101°11′42″ E). In August 2022, six samples were collected from each sampling area, for a total of 30 samples. Soil was carefully removed from the surface of the samples and placed in liquid nitrogen for temporary storage. All samples were immersed in 70% ethanol for 5 min, 2.5% NaClO solution for 2 min, and 70% ethanol for 1 min, then rinsed five times in sterile water [19,20].

### 4.2. Untargeted Metabolomics Analysis

Samples were weighed before the extraction of metabolites, and dried lyophilized samples were ground in a 2 mL Eppendorf tube containing a 5 mm tungsten bead for 1 min at 65 Hz in a grinding mill. Metabolites were extracted using 1 mL precooled mixtures of methanol, acetonitrile, and water (*v*/*v*/*v*, 2:2:1); then, they underwent 1 h ultrasonic shaking in ice baths. Subsequently, the mixture was kept at −20 °C for 1 h and centrifuged at 14,000× *g* for 20 min at 4 °C. The supernatants were recovered and concentrated to dry in a vacuum.

Metabolomics profiling was analyzed using a UPLC-ESI-Q-Orbitrap-MS system (UHPLC, Shimadzu Nexera X2 LC-30AD, Shimadzu, Japan), coupled with Q-Exactive Plus (Thermo Scientific, San Jose, CA, USA). For liquid chromatography (LC) separation, samples were analyzed using an ACQUITY UPLC^®^ HSS T3 column (2.1 × 100 mm, 1.8 μm) (Waters, Milford, MA, USA). The flow rate was 0.3 mL/min, and the mobile phase contained the following: A: 0.1% FA in water and B: 100% acetonitrile (ACN). The gradient was 0% buffer B for 2 min, and it was linearly increased to 48% at 4 min, then, it was increased to 100% at 4 min, and maintained for 2 min. Next, it decreased to 0% buffer B at 0.1 min, and a 3 min re-equilibration period was employed. The HESI source conditions were set, as follows: Spray Voltage: 3.8kv (positive) and 3.2kv (negative); Capillary Temperature: 320 °C; Sheath Gas (nitrogen) flow: 30 arb (arbitrary units); Aux Gas flow: 5 arb; Probe Heater Temp: 350 °C; S-Lens RF Level: 50. The instrument was set to acquire data over the *m*/*z* range 70–1050 Da for full MS. The full MS scans were acquired at a resolution of 70,000 at *m*/*z* 200, and 17,500 at *m*/*z* 200 for MS/MS scanning. The maximum injection time was set to 100 ms for MS and 50 ms for MS/MS. The isolation window for MS2 was set to 2 *m*/*z* and the normalized collision energy (stepped) was set as 20, 30, and 40 for fragmentation. Quality control (QC) samples were prepared by pooling aliquots of all samples that were representative of the samples being analyzed and used for data normalization. Blank samples (75% ACN in water) and QC samples were injected every six samples during acquisition.

The raw MS data were processed using MS-DIAL for peak alignment, retention time correction, and peak area extraction. The metabolites were identified via accuracy mass (mass tolerance < 10 ppm) and MS/MS data (mass tolerance < 0.02 Da), which were matched with HMDB, massbank, other public databases, and Bioprofile self-built metabolite standard library. Regarding the extracted-ion features, only the variables which had more than 50% of the nonzero measurement values in at least one group were kept.

R (version 4.0.3) and R packages were used for all multivariate data analyses and modeling. Data were mean-centered using Pareto scaling. Models were built on principal component analysis (PCA), orthogonal partial least-squares discriminant analysis (PLS-DA), and partial least-squares discriminant analysis (OPLS-DA). All the models evaluated were tested for overfitting using permutation test methods. The descriptive performance of the models was determined via R^2^X (cumulative) (perfect model: R^2^X (cum) = 1) and R^2^Y (cumulative) (perfect model: R^2^Y (cum) = 1) values, while their prediction performance was measured using Q2 (cumulative) (perfect model: Q2 (cum) = 1) and a permutation test (n = 200). The permuted model should not be able to predict classes; R2 and Q2 values at the Y-axis intercept must be lower than those of Q2 and the R2 of the non-permuted model. OPLS-DA allowed the determination of discriminating metabolites using the variable importance on projection (VIP). The VIP score value indicates the contribution of a variable to the discrimination of all the classes of samples. Mathematically, these scores were calculated for each variable as a weighted sum of squares of PLS weights. The mean VIP value is 1, and usually, VIP values over 1 are considered significant. A high score indicates a strong discriminatory ability, and thus, it constitutes a criterion for the selection of biomarkers. The discriminating metabolites were obtained using a statistically significant threshold for the variable influence on projection (VIP) values obtained from the OPLS-DA model and two-tailed Student’s *t*-test (*p*-value) using the normalized raw data at the univariate analysis level. The *p*-value was calculated via the one-way analysis of variance (ANOVA) for multiple group analysis. Metabolites with VIP values greater than 1.0, and *p*-values less than 0.05, were considered to be statistically significant metabolites. Fold change was calculated as the logarithm of the average mass response (area) ratio between two arbitrary classes. The identified differential metabolites were used to perform cluster analyses with the R package.

To identify the perturbed biological pathways, the differential metabolite data underwent KEGG pathway analysis using the KEGG database (http://www.kegg.jp; accessed on 15 June 2023). KEGG enrichment analyses were carried out using the Fisher’s exact test, and FDR correction for multiple tests was performed. Enriched KEGG pathways were nominally statistically significant at the *p* < 0.05 level.

### 4.3. 16S Sequence Analysis

The most appropriate total DNA extraction method was selected for the microbiome samples from various sources, whereas DNA was quantified via Nanodrop, and the quality of DNA extraction was checked using 1.2% agarose gel electrophoresis. The target sequences that could reflect the composition and diversity of bacterial flora, such as microbial ribosomal RNA or specific gene fragments, were taken as targets, corresponding primers were designed in accordance with the conserved regions in the sequences, and specific Barcode sequences of samples were added. Then, PCR amplification was performed on the variable regions of the rRNA gene (single or consecutive multiple) or specific gene fragments. PCR amplification was performed using TransGen Biotech’s Pfu high-fidelity DNA polymerase, and the number of amplification cycles was strictly controlled to keep the number of cycles as low as possible while ensuring the same amplification conditions for the same batch of samples. At the same time, a negative control was set. A negative control can detect microbial contamination of the environment and reagents. Any negative control amplified sample group with bands was not used for follow-up experiments. After the purification and recovery of the amplification products from the magnetic beads, the PCR amplification and recovery products were subjected to fluorescence quantification; the fluorescence reagent used was the Quant-iT PicoGreen dsDNA Assay Kit, and the quantification instrument used was a microplate reader (BioTek, FLx800; BioTek, Beijing, China). Based on the fluorescence quantification results, each sample was mixed using the appropriate proportions, in accordance with the sequencing volume requirement of each sample. Finally, sequencing libraries were prepared using Illumina’s TruSeq Nano DNA LT Library Prep Kit, and then, it was affixed onto the machine for high-throughput sequencing.

### 4.4. Correlation Analysis of Metabolomics and Microbiomics

Correlation analysis was performed on the detected statistically significant differential metabolites and differential bacterial genera, and the Spearman correlation coefficient was calculated. The Student’s *t*-test with a *p*-value of 1 was screened as a significantly different metabolite; the bacterial genus with a significant difference was screened using the LEfSe method, and the bacterial genus with a *p*-value < 0.05 was defined as a significant difference. In order to simplify the data of the drawing, the absolute values of the correlation coefficients between the two groups were sorted, the top 60 elements were selected, and the R (version: 4.0.5) and heatmap packages were used for subsequent drawings [20].

## 5. Conclusions

A total of 1290 metabolites and 437 differential metabolites were identified using the root samples of *B. vivipara* from different regions. Among them, flavonoids, isoflavonoids, and benzopyrans were the main medicinal components of *B. vivipara*. In addition, irigenin, an important medicinal component, was identified for the first time. There were differences between endophytic bacterial communities in the root samples from different regions. Among them, it was also found that Proteobacteria and Actinobacteriota may be the key bacterial phyla involved in the regulation of the medicinal component content in the roots of *B. vivipara*.

## Figures and Tables

**Table 1:** α-diversity index of bacterial microbial communities in different sampling areas.

Sample	Chao1	Simpson	Shannon	Good’s Coverage
GD_1	322.661	0.488432	1.48113	0.999039
GD_2	782.503	0.499047	2.43928	0.997733
GD_3	411.038	0.400322	1.49202	0.999205
GQ_1	218.223	0.406927	1.17572	0.999249
GQ_2	307.265	0.452603	1.44211	0.999244
GQ_3	157.634	0.493170	1.14908	0.999448
HL_1	1803.710	0.820674	5.42361	0.995588
HL_2	3341.240	0.969945	8.77754	0.994663
HL_3	2368.240	0.895206	6.71797	0.994931
XH_1	967.941	0.623918	3.17114	0.999507
XH_2	1361.050	0.609018	3.55784	0.998728
XH_3	1339.260	0.655782	3.81046	0.997838
ZD_1	1751.590	0.901730	6.51395	0.996411
ZD_2	1746.330	0.966805	7.55924	0.998378
ZD_3	2269.150	0.990300	8.67914	0.997106

## Data Availability

Data are contained within the article.

## Supplementary Materials

The following supporting information can be downloaded at: https://www.mdpi.com/article/10.3390/molecules29040860/s1, Figure S1: Dilution curves of endophytic bacterial communities in *B. vivipara* roots; Table S1: Identification of all metabolites and their positive and negative ion patterns in the root of *B. vivipara*; Table S2: Screening for differential metabolites in the root of *B. vivipara*.
