# Supplementary figures and images for: Revealing Medicinal Constituents of Bistorta vivipara Based on Non-Targeted Metabolomics and 16S rDNA Gene Sequencing Technology
Source: Molecules. 2024 Feb 15;29(4):860. doi: 10.3390/molecules29040860 (PMC10892765; doi:10.3390/molecules29040860)

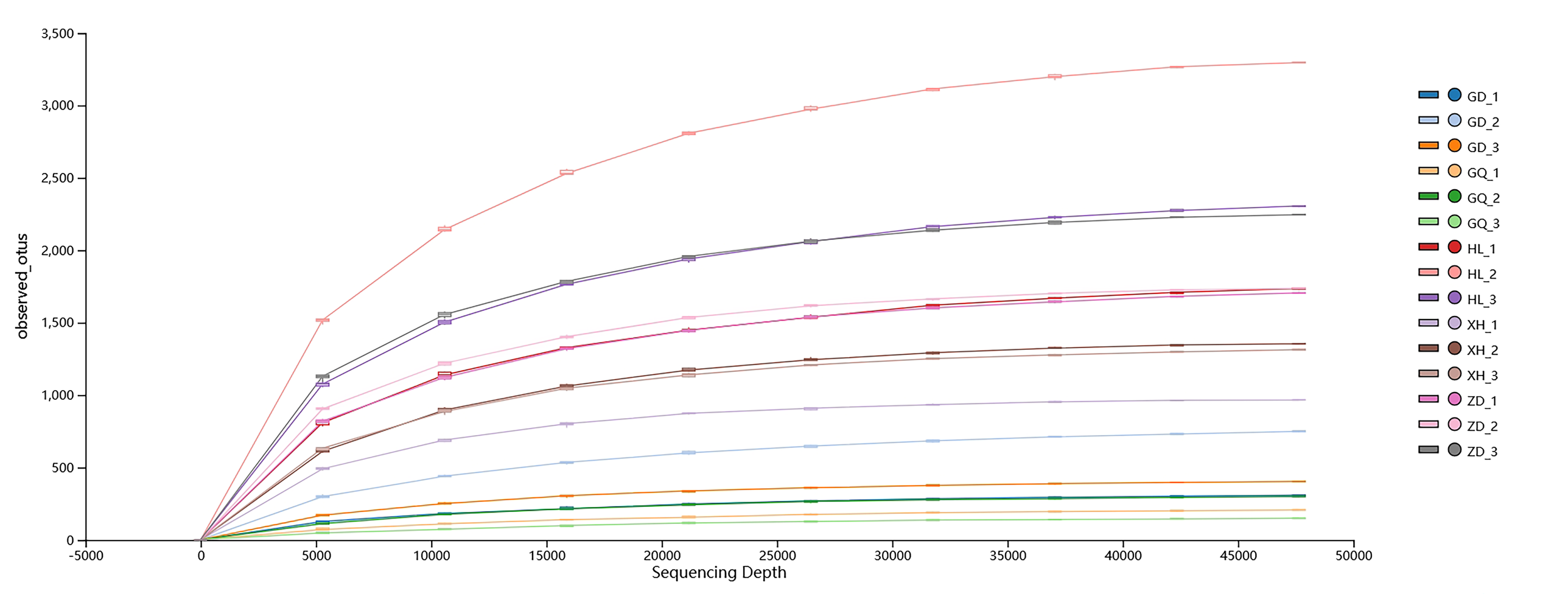

Supplement: Supplementary file 1 [file molecules-29-00860-s001.zip › Figure S1 Dilution curves of endophytic bacterial communities in B. vivipara roots..png]
